# “It’s Unjust to Treat Pregnant People this Way”: Obstetric Violence Among Pregnant and Postpartum Asylum-seekers at the Mexico-U.S. Border

**DOI:** 10.1007/s10903-026-01857-4

**Published:** 2026-02-12

**Authors:** Shira Goldenberg, Isela Martinez SanRoman, Kaylee Ramage, Nicole Elizabeth Ramos, Ietza Bojorquez

**Affiliations:** 1 San Diego State University, San Diego, USA; 2 San Diego State University Research Foundation, San Diego, USA; 3 University of Tennessee at Knoxville, Knoxville, USA; 4 Al Otro Lado, San Ysidro, USA; 5 El Colegio de la Frontera Norte, Tijuana, Mexico

**Keywords:** Reproductive rights, Obstetric violence, Asylum seekers, Women’s health, Mexico-U.S. border, Immigration policy

## Abstract

To describe asylum-seeking women’s perspectives on and lived experiences of obstetric violence, including how it is shaped by structural contexts related to stigma, discrimination, and migration policies and systems. We conducted a qualitative study involving thematic analysis of in-depth interviews conducted July 2022-April 2023 with pregnant and postpartum asylum-seeking women at the Mexico-U.S. border (*N* = 38). Asylum-seeking pregnant and postpartum women faced obstetric violence across the migration process and within both health and immigration systems. This was experienced in hospitals, immigration detention, and other clinical and non-clinical settings and included denial of timely and responsive care; discriminatory, disrespectful and dehumanizing treatment; physical and verbal abuse; and lack of informed consent for procedures. Women described obstetric violence as frequent, with some attributing this to intersectional stigma and discrimination. Obstetric violence represents a severe violation of the human and reproductive rights of pregnant and postpartum asylum-seekers which requires urgent policy action and intersectoral interventions. Culturally-appropriate, trauma-informed reproductive healthcare, provider sensitivity trainings, changes to immigration policies, and anti-discrimination interventions within and beyond the health system are recommended.

## Background

The number of forced migrants continues to increase worldwide [1], with women representing half of the estimated 117.3 million displaced people in 2023 [2]. Amid rising displacement driven by political instability, violence, poverty and natural disasters [3], asylum-seekers face myriad structural challenges during long, arduous, and unpredictable migration journeys [4]. Asylum-seekers who migrate while pregnant or postpartum face challenges in perinatal and mental health, as well as risks of maternal mortality, preterm birth, congenital anomalies, and barriers to perinatal care [5]. In recent years, rising asylum deterrence policies and anti-immigrant rhetoric have exacerbated threats to asylum-seekers’ health [4].

Obstetric violence (OV) represents a violation of human rights with serious health and social implications [6]. This includes physical, sexual, or verbal abuse (e.g., dehumanization, threats, scolding, humiliation) during childbirth; performing clinical procedures without informed consent; failure to provide timely and responsive care; and other forms of disrespectful or neglectful treatment [7–9]. These violations are driven by structural inequities including patriarchal gender norms, gender-based violence, lack of respect for reproductive rights, and health system constraints [9, 10]. In addition to violating reproductive and human rights, OV is associated with adverse short- and long-term health consequences including breastfeeding problems, poor self-rated health, post-traumatic stress, postpartum depression, health system mistrust, and unwillingness to access care [11].

OV is a recognized threat to perinatal health within low and middle-income countries, particularly in Latin America [7, 12, 13]. Yet, limited work has examined experiences of OV among asylum-seekers [7], or over the migration process. Asylum-seekers face gaps in access to affordable, accessible, and dignified healthcare, and mistreatment within health and immigration systems in many transit and receiving contexts [14, 15]. OV may be compounded by unique risks encountered by asylum-seekers during migration, yet few prior studies have investigated this, particularly in complex border settings. A scoping review found that migrant women and girls in Mexico and Central America encounter considerable barriers to sexual and reproductive health services, with particularly limited information available on reproductive health including OV [16]. Whereas qualitative work has documented how structural threats during transit and at the Mexico-U.S. border undermine reproductive health and rights of asylum-seeking women [17], to our knowledge no previous binational studies have investigated OV among asylum-seekers at the US-Mexico border.

The U.S. is the top destination for Latin American and Caribbean asylum-seekers [18, 19]. Mexico-U.S. border cities represent major transit and destination routes for asylum-seekers [19]. The past decade has witnessed unprecedented implementation of policies that deter asylum-seekers, including ‘Title 42,’ a longstanding public health policy used to expel migrants during the COVID-19 pandemic [20, 21]. The ongoing arrival of migrants, coupled with implementation of such policies, has posed challenges to already-stretched health and social infrastructure in Mexican border cities [19], particularly amid the COVID-19 pandemic [22]. Amid this context, we aimed to examine asylum-seeking women’s perspectives on and lived experiences of OV and how these are shaped by structural factors and contexts across health and immigration systems.

## Theoretical/Conceptual Framework

This study drew on theoretical principles of structural violence [23, 24] and the multi-staged determinants of migrant health [25] to conceptualize the ways in which structural factors across migration and health systems can influence OV. In this study, we operationalized structural violence as “invisible” or “indirect” violence in which inequitable systems and social structures cause preventable suffering, morbidity, or mortality [23, 24]. We use this as a lens for examining the broader social, economic, and political forces and systems that serve as “causes of the causes of poor health.” [23] We apply this frame to analyze the experiences of obstetric violence amongst marginalized migrant women – in this case, asylum-seekers – who face interlocking threats to health related to stigmatization, precarious living conditions, and immigration policies and practices that hinder their access to health and minimize their sense of entitlement to such rights [26]. Additionally, we drew on the multi-staged determinants of migrant health [25] framework to examine how these experiences and their drivers may change over the migration process.

### Methods

#### Participants

We analyzed qualitative data collected with asylum-seeking women^1^ (*N* = 38) in Tijuana, Mexico and multiple sites in the U.S. between July 2022-April 2023 as part of a binational, academic-community collaboration known as the Migration-Related Inequities in Health among Refugee & Asylum-Seeking Women (MIHRA) project [27]. Participants were pregnant or postpartum women of reproductive age (18–49), migrated to the Mexico-U.S. border for the purpose of seeking asylum in the U.S., and able to provide informed consent. Recruitment was via posters, word-of-mouth, direct referral, and outreach in collaboration with a community-based partner organization that serves migrants in Tijuana, Mexico and the U.S. Women were purposively sampled to reflect diverse experiences, including migration history (e.g., country, duration) and age. Interviews were conducted in-person in Tijuana or remotely with those in the U.S.

## Data Collection and Measures

As previously described [27], following informed consent, in-depth interviews were conducted in Spanish or in Haitian Creole with a trained interpreter. Data collection was by bilingual staff trained in trauma-informed interviewing. Recruitment and interviews were led by team members who identified as women and had either personal migration experience in the U.S.-Mexico binational context or were intimately familiar with the local community context and challenges faced by migrants and asylum-seekers. Trauma-informed data collection involved approaching the recruitment and interview process with an understanding that past trauma can have a substantial impact on what is shared, how comfortable participants feel sharing, and the services and supports they may need. Interviewers were trained not to press participants to disclose information they did not appear comfortable sharing; to take time to establish rapport with participants; to take breaks or move on in the interview when needed; and to provide extensive referrals to health, social, migration, and other services to support participants’ needs. The team’s positionality, expertise and training facilitated high levels of rapport during the interview process.

A brief sociodemographic survey was administered before the interview. Interviews were audio-recorded and ~ 60–90 min. Sample topics and questions from the interview guide that were used in the current analysis are included in Table 1. Participants received extensive referrals and an honorarium or gift card of $30 USD. Procedures were approved by Institutional Review Boards in the U.S. and Mexico.

**Table 1:** MIHRA study semi-structured interview guide topics and sample questions and probes

Topic	Sample Question & probes
Migration Experiences	Could you tell me about how and why you arrived here and your reasons for seeking safety in the U.S.? Sample probes: *● *Can you describe some of the reasons you’ve come here? *● *Who did you come here with? *● * How long have you been living or working here?
Experiences with reproductive health and health care services	Can you tell us about your experiences with accessing reproductive health services for pregnancy and childbirth during your migration journey? Or any challenges you faced to getting the care you needed? Sample probes: *● *Think about the pregnancies you have had since the pandemic began. When and how did you find out that you were pregnant? Where did this occur (before or after your migration)? *● *Did you seek prenatal, intrapartum, and/or postpartum care? When? *● *What healthcare services were offered to you and by whom?

### Data Analysis

Interviews were transcribed, translated, accuracy checked and de-identified by bilingual team members. The initial codebook was developed based on the study’s interview and research questions, as well as emergent codes identified based on participants’ narratives and feedback from community partners, and iteratively revised as data collection and analyses progressed. Coding of interviews was completed by multiple team members. Analysis for this study was led by the second author and began with an iterative, team-based process of reading, discussing, and coding in collaboration with interdisciplinary team members and community partners via biweekly meetings. We conducted inductive thematic analysis [28] to identify experiences of OV across health and immigration systems and analyze the ways in which structural factors influenced experiences of OV and mistreatment. In the final stage of analysis, our interpretation of the data explicitly drew on the theoretical lens of structural violence [23] and the multi-staged determinants of migrant health [25] to examine the structural origins of OV faced by asylum-seekers across both health and immigration systems, and potential variations in these experiences over the migration process.

## Results

Of 38 participants, 29 were interviewed in Tijuana and 9 in the U.S. (Table 2). Participants’ average age was 28.7 years. Primary countries of origin were Mexico and Honduras, followed by Haiti, El Salvador, and Guatemala. The mean number of children was 2.3, and the mean duration of time participants spent in Tijuana waiting to cross the border was 10.7 months.

**Table 2:** Sociodemographic characteristics of asylum-seeking women, MIHRA study, 2022–2023 (*N* = 38)

Characteristic	*n* (%) or mean (range)
**Interview location**	
Tijuana, Mexico	29 (76.3%)
U.S.	9 (23.7%)
**Age**, *in years*	28.7 (18– 41)
**Country of Origin**	
* Mexico*	13 (34.2%)
* Honduras*	10 (26.3%)
* Haiti*	6 (15.4%)
* El Salvador*	5 (13.2%)
*Guatemala*	4 (10.2%)
**Race/ethnicity**	
* Indigenous*	8 (21.1%)
* Hispanic/Latina*	33 (86.8%)
* Black*	8 (21.1%)
**Number of Children**	2.3 (1– 5)
**Duration in Tijuana**, *in months*	10.7 (1–72)
**Language most comfortable with**	
* Spanish*	29 (76.3%)
* Haitian Creole*	6 (15.8%)
* Other*	3 (7.9%)

Women’s narratives indicated that they experienced myriad forms of obstetric violence during migration, with instances reported during migration both while transiting Mexico and in the U.S OV emerged as a severe example of the more generalized mistreatment faced by many – though not all - migrant mothers. OV was experienced across both health and immigration systems, including within public hospitals, in U.S. immigration facilities, and transit communities in Mexico and Central America. The types of OV experienced included denial of timely and responsive care; discriminatory, disrespectful and dehumanizing treatment; physical and verbal abuse; and lack of informed consent for procedures (Table 3). Perpetrators were primarily healthcare providers, clinic staff, and immigration authorities. In contrast, participants who received care from a community-based organization offering midwifery support in Tijuana described having dignified or respectful birth experiences.

**Table 3:** Examples of types of obstetric violence experienced by asylum-seeking women across health and immigration systems, MIHRA study (2022–2023)

Type of Obstetric Violence	Participant Example
Physical abuse	*They left me alone and turned the lights off…Every time they came in*,* they would put their hand inside me…I was so afraid.* *(El Salvador*,* Age 29)*
Verbal abuse	*He yelled at me pretty badly. I told him “I am telling you that my baby is coming and you say no*,* [but] I am the one who feels [it].” [He said something derogatory] that I did not like.* *(Honduras*,* age 26)*
Failure to provide timely and responsive care	*They wouldn’t listen to me…they were laughing at me because I was in pain.* *(*Guerrero, MX, 23) *Interviewer: Were you offered pain medication at any time?* *Participant: No*,* not at all. This is something normal and even more so when you already have two children*,* three*,* you know*,* you know*,* I mean*,* you have nothing to complain about anymore*,* you are not even a first timer.* *(*Michoacan, MX, age 30) *I woke up bleeding*,* so I went to the hospital*,* and they checked me there and told me that it was not yet time… “Then why am I bleeding?” “No*,* go home and come back when it’s time.” … I asked the doctor if he wanted to see my ultrasounds. I mean*,* I am not going to order the doctor [to do his job] maybe I could have had a normal [birth]*,* but […] If I didn’t show my ultrasounds*,* this child might have died in my belly.* *(Guatemala*,* Age 24)*

### ‘*I’m Afraid of [Dying] in the Hospital’*: Obstetric Violence within Healthcare Settings During Migration

Most participants who had given birth during the migration process did so in public hospitals in Mexico. There, many experienced OV that included verbal or physical abuse, discriminatory and dehumanizing treatment, lack of informed consent for procedures, lack of respect for patient preferences, limited provision of information, and disrespectful and dismissive treatment of patients’ family members. In some cases, this also included reproductive coercion, withholding of pain medication, and physical or sexual abuse.

Women’s accounts of OV during pregnancy, labor and delivery frequently involved accounts of discrimination by healthcare providers, which was perceived as related to intersecting identities defined by migration status, class, race/ethnicity, pregnancy, poverty, and health insurance. Participants frequently attributed adverse experiences including failure to receive timely and responsive care, reproductive coercion, lack of informed consent for procedures, and denial of needed medical care to discrimination:

*People are taken care of very badly because [healthcare providers] are very racist against black people…They look at them badly. To this day*,* Haitians are not taken care of. Haitians who are pregnant*,* they say “no*,* there is no doctor.”*

-Haiti, Age 23.

Disrespectful and abusive treatment of pregnant women and migrants in public hospitals and clinics was perceived to be widespread. For some, this was normalized to the extent that women internalized instances of clearly abusive or disrespectful care as ‘fine’ and ‘just how it is’. Such narratives often occurred within a context of accessing, or attempting to access, perinatal services within overburdened, under-resourced systems. Numerous women were turned away by providers, forced to wait outside clinics and hospitals for long durations, and had their clinical needs neglected until the very last moments of labor. As a result of anticipated discrimination and other healthcare barriers, pregnant and postpartum women frequently avoided or reduced contact with health services during migration, resorting to self-care practices or neglecting their needs altogether:

*I went home and rested because I was aware that they were not going to help me. Even the people here asked me*,* “Aren’t you about to give birth soon?” […] [I was warned] to be careful because the baby of an acquaintance of mine was left to die. They said that they didn’t want to take care of her just because she wasn’t Mexican*,* just because she was an immigrant*,* they let her baby die.*

-El Salvador, Age 21

*I took my own stitches out [because] I was afraid they wouldn’t treat me well.*

-El Salvador, Age 29.

Some experienced verbal and physical abuse during labor and delivery, as the testimonial of a woman whose baby nearly died during childbirth illustrates:

*He [the doctor] did not want to help me. My baby was out already. And he grabbed him and put him back in me… that doctor…my baby. On the stretcher. He put him back in. And that well*,* that stayed with me. Obviously*,* they are not going to write that down anywhere*,* right?*

-Honduras, Age 26.

Birth trauma and unmet medical needs were exacerbated by and contributed to deep mistrust of the health system, with numerous women describing themselves as at the mercy of an inherently discriminatory, hostile system. Women reported feeling particularly vulnerable to abuse when they had to navigate labor and delivery alone, as was often the case due to hospital rules restricting accompaniment and family/partner communications during labor and delivery.

### ‘At the Hospital, the Immigration Agents were Harassing me:’ Obstetric Violence in the Context of Immigrant Processing and Detention

Numerous participants in the U.S. shared examples of OV experienced within immigration processing and detention, including severe verbal and physical abuse and dehumanizing treatment. Most had endured long, difficult migration journeys and unpredictable waiting times to seek asylum in the U.S., during which they survived myriad physical and mental stressors while pregnant, including food insecurity and lack of safe shelter:

*Ay*,* you should have seen when I arrived there*,* how terrible it was [at the shelter][…] it was sad to sleep on the floor and I was…my belly was bigger. Ay*,* a horrible pain in the back […] maybe once a day*,* I ate*,* and I got so thin.*

-Honduras, Age 37.

Women described facing degrading, dehumanizing, and sometimes abusive treatment and denial of care by immigration officers and some health providers during immigration processing and detention. Women often attributed this to them being the targets of border and law enforcement authorities whose vitriol towards migrants – particularly pregnant migrants – was enacted upon them:

*They [police officer] grabbed me very hard*,* from here*,* from the neck. He threw me against the floor*,* against the ground. He was very tall. He yelled*,* “bitches!” at us. He called us “damned bitches”! […] He grabbed me from here*,* threw me to the ground. I put up my hands so as not to fall with my stomach. He saw that I was pregnant. He threw me down. The impact was so strong that it left me aching. I was left in a lot of pain […] I needed a doctor to check me because*,* well*,* I was in a lot of pain. Then they told me that I was causing drama and that I just wanted my son to be born there […] They refused to let me see a doctor.*

-El Salvador, Age 28.

Following her immigration processing, this participant’s requests for medical assistance were denied, and she instead was immediately expelled to Mexico.

Pregnant and postpartum women who had entered the U.S. described experiencing verbal and physical abuse during their processing at ports of entry, including being yelled at or in some cases physically abused. Those who gave birth in immigration custody described birthing conditions and processes as disrespectful and dehumanizing, and were often deported very soon after, without resources or supports to ensure their safety or wellbeing:

*“At no point were you advised to leave your country in this state*,*” [the officer] told me. I felt that he treated me very badly. The truth is that I started to cry*,* and I felt like my world was falling apart. I was [very] close to getting on my knees and begging him not to deport me with my baby.*

-Honduras, Age 29

*I was so exhausted from my delivery […]the immigration people came in… it hadn’t even been half an hour [since delivery][…]. Then the immigration agent brought his computer. “We are going to take your picture.” They took my picture and he said*,* “Sign these [removal] documents.“… when I left the hospital*,* I went out in my hospital gown.*

Interviewer: *Did they not provide you with clothing?*

*No. The [Mexican] consul was the one who saw me like that and said*,* “Mija I can’t take you over the bridge like this.”*

-Guerrero, MX, Age 27.

In one case, a woman described being so frightened while interacting with immigration officers that she went into early labor while being processed at the port of entry:

*When we entered immigration*,* they treated us very badly… They were angry and mostly because I was pregnant […] The immigration lady said it was a bad habit to send pregnant women […] she yelled loudly and I got scared. At that moment*,* my labor pains started.*

-Guatemala, Age 22.

Shortly after, this participant delivered her baby in a storage room in U.S. immigration before an ambulance arrived. She was transferred to a hospital, where the next morning, the doctor who treated her demanded a large payment, threatening to jeopardize her legal process in the U.S. Six months later, she still had been unable to obtain a U.S. birth certificate for her baby, for whom she was unable to secure vaccines and other care.

The challenges of being an asylum-seeker without secure immigration status, combined with lack of familiarity with English and the local context, resulted in further traumatic encounters with the healthcare system for pregnant and postpartum women, as most had not been connected to high-quality, trustworthy, or accessible primary or specialist care following arrival.

## Discussion

### New Contribution To the Literature

In this study, asylum-seeking women at the Mexico-U.S. border faced pervasive experiences of OV - a serious, under-addressed reproductive and public health concern. OV was experienced within both health and immigration systems, highlighting the ways in which it operates as a form of structural violence that requires inter-sectoral, structural interventions. Findings build on research highlighting the widespread nature of OV in Mexico [29] and provide unique data indicating that amid asylum-deterrence policies such as those enacted during the COVID-19 pandemic, pregnant and postpartum asylum-seekers at the Mexico-U.S. border faced abuse and mistreatment during labor and delivery across both health and immigration settings, which represent grave abuses of human and reproductive rights and clear manifestations of structural violence. This study is one of few to examine OV faced by asylum-seeking women. Findings are consistent with evidence highlighting the barriers to reproductive health services faced by migrant women regionally [16]. They complement existing studies which have identified discrimination and racism, poor provider relationships, and negative clinical interactions as barriers to perinatal healthcare for refugees and asylum-seekers; [5] and build on existing evidence that punitive policies and practices, including those which neglect and mistreat pregnant immigrants in detention facilities, undermine reproductive justice [30].

OV experienced by asylum-seeking women within immigration and healthcare settings while waiting or in the process of entering the U.S. highlights the adverse health impacts of restrictive immigration policies and practices, such as those governing detention of pregnant and postpartum women and families, across the migration process. Emerging data suggest that the health-related harms of restrictive immigration policies and practices perpetuate trauma [31] and are particularly borne by women of reproductive-age and their children [4]. OV was linked to anticipated and enacted discrimination experienced across both health and immigration settings, which pose severe barriers to healthcare engagement. These findings build on broader work which has highlighted the role of discrimination within health systems in Latin America and the Caribbean in undermining access to care among migrants, particularly among forced and irregular migrants [32].

This study has limitations. Many participants’ narratives referred to births during the COVID-19 pandemic, during which local health systems faced enormous strain and unprecedented migrant expulsions occurred. However, narratives primarily emphasized the deep-rooted nature of discrimination and OV towards migrants, racial/ethnic minorities, people of lower socioeconomic status, and pregnant people, not as primarily resulting from the effects of pandemic policies and health system responses. Finally, this qualitative study elicited the narratives of a purposively selected group of Latin American and Caribbean asylum-seeking women at the Mexico-U.S. border. While findings are of relevance to other similar cultural and border contexts, they may not be fully generalizable.

Findings suggest a critical need for policy and practice changes to address OV. Policy shifts away from asylum deterrence towards ensuring opportunities for safe, orderly migration and the right to seek asylum are needed. Fostering more inclusive immigration policy environments is vital for reducing discrimination towards asylum-seekers and promoting safer environments for labor and delivery during migration. Within immigration facilities, recommendations and required actions regarding pregnant, postpartum, nursing individuals, and infants in custody have been published in recent years in response to growing concerns [33], including “offering all pregnant persons medical assessments at CBP facilities with onsite medical support.” The implementation gaps identified by our study require urgent action. Within health systems, scaling-up culturally appropriate, trauma-informed, and migrant sensitive training in reproductive health care services is needed [34], especially in complex border contexts. Increasing access to community-based providers, including midwives, traditional birth attendants, or local birthing centers [35, 36] is also recommended, as is ensuring access to doulas or other birth companions (e.g., family member, partner) during prenatal visits and childbirth [35, 37]. Professional associations can support these efforts via increasing provider training, awareness, supportive supervision, and criterion-based audits [38].

## Data Availability

Data for this study are not publicly available for legal and ethical reasons, as this study involves sensitive data collected with a highly criminalized and stigmatized population of marginalized women. Under our current ethical approvals, de-identified data can be made available upon reasonable request and pending ethical approval. Please submit all requests to the corresponding author.

## Footnotes

1 In this study, ‘women’ were defined as self-identified cisgender women.

## References

[1] UNHCR, Refugee Data Finder. 2023.

[2] (UNHCR). U.N.H.C.o.R., Global Trends Report*.* 2023.

[3] Diamond MB, Testa L, Novak C, Kempton-Amaral K, Porteny T, Olayo-Méndez A. A population in peril: A health crisis among asylum seekers on the Northern border of Mexico. Cambridge, MA: Harvard Global Health Institute; 2020.

[4] Stirling-Cameron E, Ramos NE, Goldenberg SM. Deterrence-based asylum policies exacerbate health inequities among women and children seeking safety at the US–Mexico border. The Lancet Regional Health–Americas; 2023. p. 24. 10.1016/j.lana.2023.100545 PMC10336237 37448452

[5] Heslehurst N, et al. Perinatal health outcomes and care among asylum seekers and refugees: a systematic review of systematic reviews. BMC Med. 2018;16:1–25. 10.1186/s12916-018-1064-0 PMC5996508 29890984

[6] Garcia LM. Obstetric violence in the united States and other high-income countries: an integrative review. Sex Reproductive Health Matters. 2023;31(1):2322194. 10.1080/26410397.2024.2322194 PMC11005882 38590127

[7] Bohren MA, et al. The mistreatment of women during childbirth in health facilities globally: A Mixed-Methods systematic review. PLoS Med. 2015;12(6):e1001847–discussione1001847. 26126110 10.1371/journal.pmed.1001847 PMC4488322

[8] Sadler M, et al. Moving beyond disrespect and abuse: addressing the structural dimensions of obstetric violence. Reprod Health Matters. 2016;24(47):47–55. 27578338 10.1016/j.rhm.2016.04.002

[9] Šimonović D. A human rights-based approach to mistreatment and violence against women in reproductive health services with a focus on childbirth and obstetric violence. New York, NY, USA: UN; 2019.

[10] Williams CR, Meier BM. Ending the abuse: the human rights implications of obstetric violence and the promise of rights-based policy to realise respectful maternity care. Sex Reproductive Health Matters. 2019;27(1):9–11. 10.1080/26410397.2019.1691899 PMC7888047 31809245

[11] Viirman F, et al. Negative childbirth experience in relation to mode of birth and events during labour: A mixed methods study. Eur J Obstet Gynecol Reprod Biol. 2023;282:146–54. 36731207 10.1016/j.ejogrb.2023.01.031

[12] Frías SM, Castro R. Mistreatment, Abuse, and Gender-Based Violence During Childbirth: A Longitudinal Analysis of Obstetric Violence in México (2011–2021)*.* Violence Against Women, 2024: p. 10778012241289426. 10.1177/10778012241289426 39429150

[13] Castro R, Erviti J. Years of research on obstetric violence in Mexico. Revista CONAMED enero-marzo. 2014;19(1):37–42.

[14] Wirtz AL, Page KR, Spiegel PB. Ensuring the right to health for migrants and refugees. Lancet HIV. 2024;11(12):e797–8. 39536775 10.1016/S2352-3018(24)00275-3

[15] Abubakar I, et al. The UCL–Lancet commission on migration and health: the health of a world on the move. Lancet. 2018;392(10164):2606–54. 30528486 10.1016/S0140-6736(18)32114-7 PMC7612863

[16] Garbett A, et al. The paradox of choice in the sexual and reproductive health and rights challenges of south-south migrant girls and women in central America and mexico: a scoping review of the literature. J Migration Health. 2023;7:100143. 36568827 10.1016/j.jmh.2022.100143 PMC9768374

[17] Ramage K, et al. When you leave your country, this is what you’re in for: experiences of structural, legal, and gender-based violence among asylum-seeking women at the Mexico-US border. BMC Public Health. 2023;23(1):1699. 37659997 10.1186/s12889-023-16538-2 PMC10474729

[18] Moslimani M, Passel J. What the data says about immigrants in the U.S*.* 2024, Pew Research Center.

[19] Alba F. Mexico at a crossroads once more: emigration levels off as transit migration and immigration rise. Migration Policy Institute; 2024.

[20] Gostin LO, Friedman EA. *T*itle 42 exclusions of asylum Seekers—A misuse of public health Powers. In JAMA health forum. American Medical Association; 2023. 10.1001/jamahealthforum.2023.0078 36656601

[21] Fabi R, Rivas SD, Griffin M. Not in our name: The disingenuous use of public health as justification for title 42 expulsions in the era of the migrant protection protocols. 2022, American Public Health Association. pp. 1115–1119. 10.2105/AJPH.2022.306887 PMC9342824 35737927

[22] Bojorquez-Chapela I, et al. In-Transit migrants and asylum seekers: inclusion gaps in mexico’s COVID-19 health policy response: study examines public health policies developed in Mexico in response to COVID-19 and the impact on in-transit migrants and asylum seekers. Health Aff. 2021;40(7):1154–61. 10.1377/hlthaff.2021.00085 34228514

[23] De Maio F, Ansell D. As natural as the air around us: on the origin and development of the concept of structural violence in health research. Int J Health Serv. 2018;48(4):749–59. 30092699 10.1177/0020731418792825

[24] Farmer P. On suffering and structural violence: A view from below. Daedalus. 1996;125(1):261–83.

[25] Acevedo-Garcia D, et al. Integrating social epidemiology into immigrant health research: a cross-national framework. Soc Sci Med. 2012;75(12):2060–8. 22721965 10.1016/j.socscimed.2012.04.040

[26] Larchanché S. Intangible obstacles: health implications of stigmatization, structural violence, and fear among undocumented immigrants in France. Volume 74. Social science & medicine; 2012. pp. 858–63. 6. 10.1016/j.socscimed.2011.08.016 22000263

[27] Ramage K, et al. In the end… I got pregnant. And that wasn’t what the plans were: threats to reproductive health and rights for migrant women intending to seek safety in the US. SSM-Qualitative Res Health. 2025;7:p100553.

[28] Kiger ME, Varpio L. Thematic analysis of qualitative data: AMEE guide 131. Med Teach. 2020;42(8):846–54. 32356468 10.1080/0142159X.2020.1755030

[29] INEGI. Encuesta Nacional sobre la Dinámica de las Relaciones en los Hogares (ENDIREH)* 2021*. 2022.

[30] Messing AJ, Fabi RE, Rosen JD. Reproductive injustice at the US border. Am J Public Health. 2020;110(3):339–44. 31944845 10.2105/AJPH.2019.305466 PMC7002961

[31] Silverstein MC, et al. Continued trauma: A thematic analysis of the Asylum-Seeking experience under the migrant protection protocols. Health Equity. 2021;5(1):277–87. 34095707 10.1089/heq.2020.0144 PMC8175263

[32] Cabieses B, et al. Challenges for addressing migrant health in Chile during the Covid-19 pandemic: a scoping review. Salud pública De México. 2024;66(2):191–7. 39977035 10.21149/15329

[33] U.S. Customs and Border Protection. Policy statement and required actions regarding Pregnant, Postpartum, nursing Individuals, and infants in custody. 2022, U.S. Customs and Border Protection: United States.

[34] DeAndrade S, et al. Trauma-Informed care training in U.S. And Canadian Ob/Gyn residencies. Violence against Women. 2024;0(0):10778012241230328. 10.1177/10778012241230328 38356282

[35] van der Waal R, et al. Obstetric violence: an intersectional refraction through abolition feminism. Feminist Anthropol. 2023;4(1):91–114.

[36] Vedam S, et al. The giving voice to mothers study: inequity and mistreatment during pregnancy and childbirth in the united States. Reproductive Health. 2019;16(1):77. 31182118 10.1186/s12978-019-0729-2 PMC6558766

[37] WHO. The prevention and elimination of disrespect and abuse during facility-based childbirth: WHO statement. World Health Organization; 2014.

[38] Miller S, Lalonde A. The global epidemic of abuse and disrespect during childbirth: History, evidence, interventions, and figo’s mother–baby friendly birthing facilities initiative. Int J Gynecol Obstet. 2015;131(S1):S49–52. 10.1016/j.ijgo.2015.02.005 26433506

